# Feasibility of Sentinel Lymph Node Biopsy in Early-Stage Epithelial Ovarian Cancer: A Systematic Review and Meta-Analysis

**DOI:** 10.3390/diagnostics13203209

**Published:** 2023-10-14

**Authors:** Georgia Zachou, Gabriella Yongue, Dhivya Chandrasekaran

**Affiliations:** 1 Department of Surgical Gynaecological Oncology, University College London Hospital, London NW1 2BU, UK; 2 Department of Obstetrics and Gynaecology, Barnet Hospital, Royal Free London NHS Foundation Trust, London EN5 3DJ, UK

**Keywords:** sentinel lymph node, early-stage ovarian cancer, systematic review, meta-analysis

## Abstract

Sentinel lymph node biopsy (SLNB) has been widely adopted in the management of early-stage gynaecological cancers such as endometrial, vulvar and cervical cancer. Comprehensive surgical staging is crucial for patients with early-stage ovarian cancer and currently, that includes bilateral pelvic and para-aortic lymph node assessment. SLNB allows the identification, excision and pathological assessment of the first draining lymph nodes, thus negating the need for a full lymphadenectomy. We systematically searched the MEDLINE, Embase and Cochrane Central Register of Controlled Trials (CENTRAL) databases (from inception to 3 November 2022) in accordance with Preferred Reporting Items for Systematic Reviews and Meta-Analysis (PRISMA). Our search identified 153 articles from which 11 were eligible for inclusion. Patients with clinical stage I–II ovarian cancer undergoing sentinel lymph node biopsy were included. Statistical analysis was performed in RStudio using the meta package, where meta-analysis was performed for the detection. The risk of bias was assessed using the Quality Assessment of Diagnostic Accuracy Studies C (QUADAS-C) tool. Overall, 11 observational studies met the predetermined criteria and these included 194 women. The meta-analysis showed that the detection rate of sentinel lymph nodes in early-stage ovarian cancer was 94% (95% CI of 86% to 1.00%). Significant heterogeneity was noted among the studies with Q = 47.6, *p* < 0.0001, I^2^ = 79% and τ^2^ = 0.02. Sentinel lymph nodes in early-stage ovarian cancer have a high detection rate and can potentially have applicability in clinical practice. However, considering the small number of participants in the studies, the heterogeneity among them and the low quality of evidence, the results should be interpreted with caution. Larger trials are needed before a change in clinical practice is recommended.

## 1. Introduction

Ovarian cancer is the eighth most common cancer among women and the leading cause of death amongst gynaecological malignancies [1]. Around 17–24.3% of patients present with International Federation of Gynecology and Obstetrics (FIGO) stage 1 (disease confined to the ovary) and just 5% with FIGO stage 2 ovarian cancer (disease confined to pelvic organs but not involving lymph nodes) [2,3]. For patients with apparent early-stage disease on pre-operative imaging, complete surgical staging including full bilateral pelvic and para-aortic lymphadenectomy (except in stage 1 expansile mucinous adenocarcinomas) continues to be the primary management. Lymph node staging is not only important for prognostic information, but also for decisions regarding adjuvant therapeutic options in those who are upstaged [4].

The risk of microscopic pelvic or para-aortic lymph node metastases in apparent FIGO stages I–II disease is reported to be 14% (in the range 6.1–29.6%) [5,6]. Systematic lymphadenectomy is a complex procedure requiring advanced surgical skills. It is also associated with increased intra-operative morbidity (increased surgical time, blood loss and need for blood transfusion) and post-operative morbidity, including a 7-fold higher risk of lymphoedema and lymphocyst formation, vessel or nerve injury and reduced quality of life [7,8,9]. In advanced-stage ovarian cancer, systematic lymphadenectomy with clinically negative lymph nodes has not been shown to improve overall or progression-free survival [9]. Similarly, in early-stage ovarian cancer, systematic lymphadenectomy is only of diagnostic benefit and does not provide any survival benefit [10].

Sentinel lymph node biopsy (SLNB) is commonly used in gynaecological cancers such as breast and vulval cancer and has started to become an established approach in early-stage cervical and endometrial cancers [11,12,13,14,15,16,17]. It allows the first draining node from a cancer to be identified and undergo ultrastaging histological examination for metastatic cancer cells. Thus, SLNB aims to provide the same diagnostic information that a full lymphadenectomy would provide without the associated intra-operative risks and morbidity for a procedure that does not incur survival benefit. In parallel to developing SLNB procedures in gynaecological cancers, there has been a trend for minimally invasive approaches to managing early-stage ovarian cancer, in particular the use of laparoscopic or robotic staging in disease confined to the ovary [18,19].

Several observational studies have examined the hypothesis that SLNB could potentially provide the diagnostic and prognostic accuracy that systematic lymphadenectomy does, without a significant increase in the morbidity or surgical time. This systematic review and meta-analysis aim to evaluate the current evidence available for the role of SLNB in early-stage ovarian cancer.

## 2. Materials and Methods

### 2.1. Search Strategy and Selection Criteria

**Figure 1:**
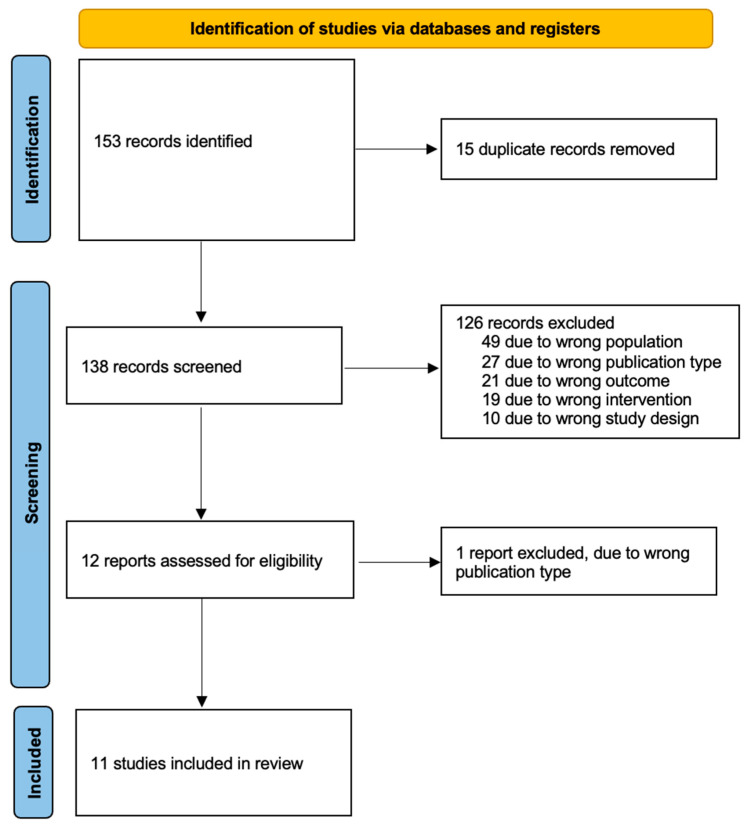
PRISMA flow diagram.

This systematic literature review was conducted according to the Preferred Reporting Items for Systematic Reviews and Meta-Analyses (PRISMA) criteria (Figure 1) [20]. A systematic literature search was performed for relevant studies from inception to 3 November 2022 across the following databases: Embase (OVID), MEDLINE (OVID), Google Scholar, ClinicalTrials.gov and the Cochrane Central Register of Controlled Trials (CENTRAL). Review articles were also hand searched for relevant studies. The following search terms were used in OVID: (“ovarian neoplasms” (MeSH terms) OR “ovarian neoplasms” (MeSH terms)) AND (“radioactive tracers” (MeSH terms) OR “coloring agents” (MeSH terms) OR “indocyanine green” (MeSH terms) OR “technetium” (MeSH terms) OR “spectroscopy, near infrared” (MeSH terms) OR “methylene blue” (MeSH terms) OR “fluorescent antibody technique” (MeSH terms)) AND (“sentinel lymph node biopsy” (MeSH terms) OR “lymph nodes” (MeSH terms) OR “lymph node excision” (MeSH terms)). The same search strategy was adapted to Google Scholar.

### 2.2. Inclusion and Exclusion Criteria

Randomised controlled trials (RCTs), cohorts and case–control studies looking at SLNB in early-stage epithelial ovarian cancer were included. Studies included patients with early-stage epithelial ovarian cancer who had undergone SLNB detection (index test). For diagnostic accuracy, the studies had to have proceeded with full pelvic and para-aortic lymphadenectomy (reference standard). The study design could have been prospective or retrospective. As we expected to find studies with small numbers of participants, all the relevant studies irrespective of the numbers of participants were included.

### 2.3. Data Extraction

Titles and abstracts retrieved by electronic searching were exported to the systematic review management tool, Rayyan (Rayyan.ai), and duplicates were removed using the “find duplicates” software tool and by manual checking [21]. Two review authors (G.Z. and G.Y.) independently assessed the eligibility of the papers. Studies that clearly did not meet the inclusion criteria were excluded, 49 of them due to the wrong population, 27 due to the wrong publication type, 21 due to the wrong outcome, 19 due to the wrong drug and 10 due to their study design. Copies of the full text of potentially relevant references were obtained. After the full-text screening, we excluded 1 study due to the wrong publication type. Any disagreements between authors were resolved by discussion or by the involvement of a third review author (D.C.).

The following data were collected for each study: first investigator’s name, publication year, country, sample size, age, body mass index, menopausal status, type of surgery, surgical approach, FIGO stage, histological type, SLNB technique, location of injection, type of tracer, number of LNs, location of LN, detection rate and intra-operative complications.

### 2.4. Outcome

The primary outcome of interest was the detection rate in order to investigate the feasibility of SLNB. The secondary reported outcomes were the intra-operative complications and the diagnostic accuracy.

### 2.5. Assessment of Risk of Bias in Included Studies

The quality of the studies was assessed with the “Quality Assessment of Diagnostic Accuracy Studies” (QUADAS-C) tool [22]. This tool assesses four domains: patient selection, index test, reference standard, and flow and timing. The risk of bias was judged as “low”, “high” or “unclear” in each domain.

### 2.6. Statistical Analysis

The Hartung, Knapp, Sidik and Jonkman (HKSJ) approach was used to calculate the detection rates (proportion) and their 95% confidence intervals (CI) instead of the DerSimonian and Laird (DL) method for random effects meta-analysis using the metafor package in RStudio [23]. The HKSJ method seems to outperform the DL method, resulting more adequate error rates [24]. The statistical heterogeneity was assessed using the I^2^ statistics and Cochrane Q tests. Subgroup analyses were not performed in view of the limited number of cases in each study and the underreporting of demographic parameters. The possibility of publication bias was assessed visually by funnel plot and formally by the Egger’s regression test for funnel plot asymmetry using RStudio [25].

## 3. Results

### 3.1. Study Selection

Our initial search yielded 153 relevant studies. After duplicates were removed, a total of 138 studies remained. A total of 12 of them were deemed eligible for full-text screening from which 11 studies met our inclusion criteria and were included in our review [26,27,28,29,30,31,32,33,34,35,36] (Figure 1). All of them were prospective studies; no randomised control trial was identified. Of the included studies, three of them were conducted in Spain [27,28,30], three in Italy [29,32,33], two in the Netherlands [26,35], one in Finland [31], one in Iran [34] and one in Japan [36].

### 3.2. Quality Assessment

The quality of the studies was assessed with the QUADAS-C tool, although due to the aforementioned methodological heterogeneity, this should be interpreted with caution (Table 1). It was unclear in all studies if consecutive or random sampling of participants was enrolled. Regarding the index and the reference test, none of the studies were designed so that the index test results were interpreted without knowledge of the result of the reference standard, or the reference standard results interpreted without knowledge of the results of the index tests. Different surgical approaches, laparotomy, laparoscopy or robotic surgery, were adopted among and within the studies. It is unclear how this may have affected the detection of SLN (the index test) or the ability to perform full lymphadenectomy (the reference standard). These lead to unclear risk of bias for all studies about the conduct and interpretation of the index test and reference standard. There were no concerns regarding the time interval between the index and the reference standard. Most studies used full lymphadenectomy as the reference standard, except for Kleppe et al.’s, which could have introduced bias in the flow and timing domain [35]. There were no concerns regarding the applicability. 

Significant heterogeneity was noted in the demographic and methodological characteristics of the included studies (Table 2 and Table 3). Specifically, three studies used indocyanine green (ICG) and radioisotope [27,28,30], three studies used blue dye and radioisotope [26,31,35], three studies used ICG alone [29,32,33], one used radioisotope alone [34] and one used charcoal solution [36]. Also, the surgical approach to identifying sentinel lymph nodes (SLNs) was different with four studies performing laparotomy only [31,34,35,36], two studies performing laparoscopy only [32,33], two studies using a combination of laparoscopy and laparotomy [28,30] and one study using the robotic system as well [29]. For two studies, the surgical route was not available [26,27]. Patient characteristics are summarised in Table 2. There were significant differences among studies in terms of median age, body mass index (BMI), immediate or delayed surgical staging, FIGO staging and histological type.

### 3.3. Sentinel Detection

**Figure 2:**
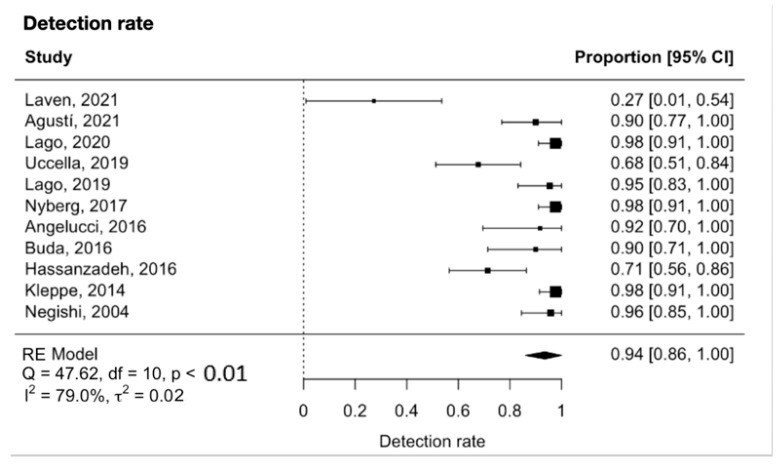
Forest plot for the detection rate [26,27,28,29,30,31,32,33,34,35,36].

**Figure 3:**
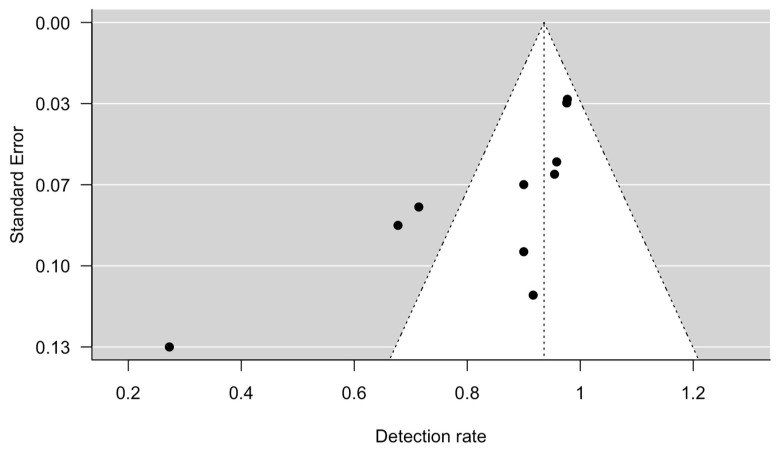
Funnel plot, using detection rate and standard error.

The meta-analysis showed that the detection rate of SLN in early-stage ovarian cancer was 0.94 (95% CI 0.86 to 1.00, Figure 2). Significant heterogeneity was noted with Q = 47.6, *p* < 0.0001, I^2^ = 79% and τ^2^ = 0.02. A funnel plot was used to visually assess for potential publication bias (Figure 3).

## 4. Discussion

Full pelvic and para-aortic lymphadenectomy is currently recommended for complete staging of early ovarian cancer as radiological methods of detecting lymph nodes are inadequate [4]. Sensitivity of radiological detection of lymph node metastasises ranges from 73.2% in positron emission tomography (PET), to 42.6% for computed tomography (CT) and 54.7% in magnetic resonance imaging (MRI) [37]. However, complete lymphadenectomy is associated with increased intra- and post-operative complications [7,8]. SLNB, if proven to be oncologically non-inferior, could have a significant impact in patient morbidity and quality of life.

In our meta-analysis, the pooled detection rate of SLNB in early-stage ovarian cancer was 94% (95% CI 86% to 100%, I^2^ = 79%, *p* < 0.0001). This is in agreement with a recent meta-analysis on lymph node detection in early-stage ovarian cancer, which included nine studies and reported a detection rate of 93.3% per patient (95% CI 77.8% to 100%; I^2^ = 74.3%, *p* < 0.0001) [38]. This is also comparable to the detection rates in other more well-established malignancies such as endometrial cancer, with a detection rate of 87%; breast cancer, with a detection rate of 97.9% with ICG; vulvar cancer, with a detection rate of 98% with blue dye and 99mTc radioisotope; and cervical cancer, with a detection rate of 98% with ICG [12,13,16,39]. It is likely that the SLNB detection rate in ovarian cancer will improve further with increased experience and refinement of technique.

### 4.1. Tracers

All the studies used one of or a combination of blue dyes, ICG or 99mTc radioisotope, except for Negishi et al.’s who used carbon nanoparticles (CH40) [36]. These tracers, apart from CH40, are commonly used in other gynaecological malignancies and are those generally recommended in national and international guidelines [40].

Blue dye, of which methylene blue is the most commonly used, has an affinity to nucleic acids and is the cheapest tracer as it does not require any additional equipment. Its visibility is not as robust as other tracers because it is not radioactive or penetrating and can therefore be easily obscured by overlying tissue [41]. Blue dye can be detected in SLNs within 15–20 min after its injection and tends to dissipate after 50 min. It has a higher rate of allergic reaction of 2% vs. 0.05% for ICG [42].

ICG is a water-soluble tricarboxycyanine which binds to albumin and fluoresces under near-infrared (NIR) light. It is highly effective, quickly absorbed and has a low toxicity. It can penetrate into tissues by 5–8 mm, allowing it to have better visibility [43]. It does, however, require extra equipment in the form of a near-infrared light source at 800 nm [41]. ICG is generally considered a more superior dye in comparison to blue dye and is the European Society of Gynaecological Oncology (ESGO)’s tracer of choice for endometrial SLNB [40]. However, it should be avoided in those with an iodine allergy or significant hepatic impairment.

99mTC binds to mannose receptors in reticuloendothelial cells found in lymph nodes. A handheld radio detector is required to locate sentinel lymph nodes intra-operatively. It has a short, six-hour half-life and a photopeak of gamma ray emission of just 140.5 keV, minimising its toxicity to patients [44]. It is also the preferred tracer for SLNB in vulvar cancer in combination with blue dye or ICG [45].

CH40 has been more commonly used in breast and gastric carcinomas. CH40 is injected as a suspension with a suspending agent and saline. Due to their size, the nanoparticles cannot enter capillaries, but instead entre the lymphatics and undergo phagocytosis by macrophages [46]. They stain the lymphatics black and have a half-life of 3 days, and no toxic side effects have been reported in the literature.

New tracers are being developed including “magnetic dyes” such as iron oxide nanoparticles, biodegradable tracers and tumour-targeted tracers. The latter are the most interesting development, as they aim to identify pathological lymph nodes without surgical removal and histopathological assessment. One such example is panitumubab-IRDye900CW. Analysis of its five most highly fluorescent lymph nodes has demonstrated 100% sensitivity, 100% negative predictive value and 85.8% specificity in identifying tumour metastases [47].

### 4.2. Tracer Injection Sites

There are three lymphatic drainage pathways described in ovarian cancer [48]. The first and most prominent is via the infundibulopelvic (IP) ligament which drains to the common iliacs bilaterally and then to the aorta on the left and the caval vein on the right. A total of 50% of patients solely drain through this route and a further 30%, in combination with the following route. The second pathway is through the ovarian ligament to the internal iliac artery plexi and the obturator fossa. A total of 20% of patients will solely drain through the ovarian ligament. Lastly, the round ligament, which drains to the inguinal nodes contributes to the rare cases of isolated inguinal metastases. Consequently, the majority of studies chose to inject their tracers into both the IP ligament and ovarian ligament stumps [26,27,28,29,30,33,34,35]. Three studies chose to inject, in some or all of their patients, into the ovarian cortex [31,32,34,36]. This technique has been criticised due to its poor detection rate, as low as 40%; the difficulty in identifying normal cortex to injected; and the risk of tumour puncture and seeding [34].

All of the studies, except two, performed immediate SLNB as opposed to delayed SLNB as part of a two-stage surgery following confirmation of malignancy on the tumour specimen. Uccella et al. found that the SLN detection rate was higher in patients undergoing immediate staging (88.9%) in comparison to those undergoing delayed staging (41.7%) [29]. These results were replicated by Laven et al., who in their small study of 11 patients, concluded that their low detection rate of just 27% was attributable to the fact that their tracers were injected after oophorectomy either at primary or secondary surgery [26]. This could be explained by the fact that lymphatic pathways are occluded and diverted following loss of perfusion of lymph fluid from the original organ. Lago et al., in both of their studies, had a 100% detection rate following injection of tracers after adnexectomy and frozen sectioning, confirming malignancy at primary surgery [28,30]. Thus, this suggests that tracers can be injected either before or after adnexectomy, but are most accurate in the primary surgical setting.

### 4.3. Ultrastaging

It is recommended that SLN undergo ultrastaging as it improves the detection of metastasis by of lymph node by 37–43% in endometrial cancer and 10–15% in cervical cancer [49]. This is due to the fact that ultrastaging identifies micrometastases and isolated tumour cells that would otherwise have gone undetected by traditional techniques. There are numerous protocols published in the literature, but generally, it involves the SLN being divided into serial sections of 50–250 μM [50]. Each slice is assessed using H&E and immunohistochemistry.

### 4.4. Strengths and Limitations

Our meta-analysis was conducted according to the PRISMA guidelines. Statistical models (HKSJ) that aim to reduce the methodological heterogeneity among the included studies were used. However, the results should be interpreted with caution due to the observational design of the included studies which affects the strength of our meta-analysis. The low quality of evidence, the small number of included studies, the sample sizes and the heterogeneity among the studies influenced the results of our meta-analysis.

## 5. Conclusions

In conclusion, SLNB holds a promising prospect for the future surgical management of early-stage ovarian cancer. Although current evidence is heterogeneous, it appears that the detection rate of ovarian SLN is comparable to that in other more established gynaecological SLNBs. However, there is not enough good quality evidence to recommend that this procedure should yet become standard practice. We look forward to receiving the results of ongoing and future trials which will add to the evidence on the sentinel detection rate as well as their diagnostic accuracy.

## 6. Future Directions

Currently, full pelvic and para-aortic lymphadenectomy is recommended for complete staging of early-stage ovarian cancer [4]. SLNB has emerged as a standard approach for staging in endometrial, cervical and vulvar cancers. Given its success in these gynaecological malignancies, there is a growing interest in investigating the feasibility and oncological accuracy of SLNB in early-stage ovarian cancer.

This systematic review and meta-analysis have evaluated the SLNB detection rate, which is one aspect that would define the success of LN mapping in early-stage ovarian cancer. Although the data are limited, the detection rate appears to be comparable to those in other gynaecological cancers in which SLNB is more established. As the studies included in this analysis have small population sizes, it was difficult to assess the sensitivity and false-negative rate of detecting lymph node metastasis, which would be crucial information prior to implementing SLNB as standard practice in early-stage ovarian cancer. Additionally, more information with regards to tracer type, injection site and mapping techniques will be welcomed.

Apropos the limited quality of evidence, larger prospective studies are needed to assess these parameters. Ongoing trials will provide vital information before the routine implementation of SLNB in early-stage ovarian cancer (NCT05184140, NCT03563781, NCT05937620, NCT05375526, NCT04051502, NCT04714931 and NCT05927818) (Table 4) [51,52,53,54,55,56,57].

## Figures and Tables

**Table 1:** QUADAS-C Bias tool.

Study	Test	Risk of Bias				Applicability Concerns			
		P	I	R	FT		P	I	R
Laven, 2021 [26]	Blue dye and 99mTc	?	?	?	✓		✓	✓	✓
Agustí, 2021 [27]	ICG and 99mTc	?	?	?	✓		✓	✓	✓
Lago, 2020 [28]	ICG and 99mTc	?	?	?	✓		✓	✓	✓
Uccella, 2019 [29]	ICG	?	?	?	✓		✓	✓	✓
Lago, 2019 [30]	ICG and 99mTc	?	?	?	✓		✓	✓	✓
Nyberg, 2017 [31]	Blue dye and 99mTc	?	?	?	✓		✓	✓	✓
Angelucci, 2016 [32]	ICG	?	?	?	✓		✓	✓	✓
Buda, 2016 [33]	ICG	?	?	?	✓		✓	✓	✓
Hassanzadeh, 2016 [34]	99mTc	?	?	?	✓		✓	✓	✓
Kleppe, 2014 [35]	Blue dye and 99mTc	?	?	?	?		✓	✓	✓
Negishi, 2004 [36]	Charcoal solution	?	?	?	✓		✓	✓	✓

P = patient selection; I = index test; R = reference standard; FT = flow and timing; ICG = indocyanine green; 99mTc = technetium-99m; ✓ indicates low risk; ✗ indicates high risk; ? indicates unclear risk.

**Table 2:** Demographic characteristics of the included studies.

Study No.	Year	Country	Study Name	Participants	Age	BMI	Menopausal Status	Surgery	Approach	FIGO Stage	Histological Type
1	2021	Netherlands	Laven et al. [26]	11	Median age: 57 (range 44–79)	N/A	Post: 7 (63.6%) Pre: 3 (27.3%) Unknown: 1 (9.1%)	Frozen section and surgical staging: 8 (72.7%) 2nd surgery for staging: 3 (27.3%)	N/A	IA: 6 (54.6%) IC: 4 (36.3%) IIC: 1 (9.1%)	Clear cell: 5 (45.5%) Εndometroid: 3 (27.2%) Serous: 1 (9.1%) Mucinous: 2 (18.2%)
2	2021	Spain	Agustí et al. [27]	20 patients with masses (8 had Ca)	N/A	N/A	N/A	N/A	N/A	N/A	N/A
3	2020	Spain	Lago et al. [28]	20	Mean age: 50 (range 35–68)	Mean BMI: 25	N/A	Frozen section and surgical staging: 11 (55%) 2nd surgery for staging: 9 (45%)	LPS: 9 (45%) LPT: 11 (55%)	IA: 7 (35%) IC: 11 (55%) IIA: 1 (5%) IIIB: 1 (5%) *	Serous: 4 (20) Endometrioid: 8 (40%) Clear cell: 5 (25%) Mucinous: 2 (10%) Other: 1 (5%)
4	2019	Italy	Uccella et al. [29]	31	Mean age: 56 (range 49–64)	Mean BMI: 25	N/A	Immediate surgical staging: 18 (58.1%) 2nd surgery for staging: 13 (41.9%)	LPS: 26 (83.9%) RBT: 4 (12.9%) LPT: 1 (3.2%)	IA: 12 (38.7%) IB: 3 (9.7%) IC3: 1 (3.2%) IIA: 4 (12.9%) IIB: 6 (19.4%) IIIA1: 2 (6.5%) * IIIA2: 2 (6.4%) * IIIB: 1 (3.2%) *	Serous: 15 (48.4%) Endometrioid: 8 (25.8%) Clear cell: 5 (16.1%) Mixed serous–mucinous: 2 (6.5%) Undifferentiated: 1 (3.2%)
5	2019	Spain	Lago et al. [30]	10	Mean age: 45 (range 26–74)	Mean BMI: 25	N/A	N/A	LPS: 3 (30%) LPT: 7 (70%)	IA: 1 (10%) IC: 5 (50%), IIA: 1 (10%) IIIA1: 2 (20%) * IIIA2: 1 (10%) *	Serous: 6 (60%) Clear cell: 3 (30%) Endometrioid: 1 (10%)
6	2017	Finland	Nyberg et al. [31]	20	Mean age: 63 (range 41–81)	Mean BMI: 26	N/A	N/A	LPS: 0 (0%) LPT: 20 (100%)	IC2: 1 (20%) IIA: 1 (20%) IIIA1: 1 (20%) IIIA2: 1 (20%) Metastatic: 1 (20%)	Benign: 11 (55%) Borderline tumour: 4 (20%) Serous: 3 (15%) Endometrioid: 1 (5%) Metastatic breast: 1 (5%)
7	2016	Italy	Angelucci et al. [32]	5	Mean age: 55 (range 43–67)	N/A	N/A	Immediate surgical staging: 5 (100%)	LPS: 5 (100%) LPT: 0 (0%)	IA: 2 (40%) IC: 2 (40%) IIB: 1 (20%)	Serous: 3 (60%) Endometrioid: 2 (40%)
8	2016	Italy	Buda et al. [33]	10 ^†^	Mean age: 51 (range 35–66)	Mean BMI: 21 (range 17–23)	N/A	Immediate surgical staging: 10 (100%)	LPS: 5 (100%) LPT: 0 (0%)	IA: 3 (43%) IC: 3 (43%) IIB: 1 (14%)	N/A
9	2016	Iran	Hassanzadeh et al. [34]	35 ^‡^	Mean age: 41 (range 16–68)	N/A	N/A	Immediate surgical staging: 35 (35%)	LPS: 0 (0%) LPT: 35 (100%)	N/A	Benign: 21 (60%) Serous: 10 (30%) Granulosa cell: 2 (6%) Mucinous: 1 (2%) Borderline: 1 (2%)
10	2014	Netherlands	Kleppe et al. [35]	21	N/A	N/A	N/A	Immediate surgical staging: 21 (100%)	LPS: 0 (0%) LPT: 21 (100%)	N/A	Benign: 7 (33%) Borderline: 8 (38%) Epithelial ovarian cancer: 6 (29%)
11	2004	Japan	Negishi et al. [36]	11	Mean age: 59 (range 49–71)	N/A	N/A	Immediate surgical staging: 11 (100%)	LPS: 0 (0%) LPT: 11 (100%)	N/A	Endometrioid endometrial: 9 (82%) Carcinosarcoma: 1 (9%) Serous: 1 (9%)

N/A = not available; Ca = cancer; LPS = laparoscopy; LPT = laparotomy; RBT = robotic surgery; * = upstaged; ^†^ = 7 had ovarian Ca and 3 had cervical Ca; ^‡^ = 13 had ovarian Ca, 21 benign and 1 borderline.

**Table 3:** Technical study characteristics and outcomes.

Study No.	Tracer Used			Location of Injection			Surgery			No. of LNs Median	Location of LN			Detection Rate	Intra-operative Complications	Comments
	Blue Dye	ICG	Tc-99m	IPL	UOL	Cortex	Primary	Secondary	Full LND		PA	PN	Both			
1	✓ 0.2 mL	–	✓ 20 MBq	✓	✓	–	✓ 8/11	✓ 3/11	0	N/A	66.7%	–	33.3%	3/11 (27%)	0/11 (0%)	No SLN detected by blue dye
2	–	✓	✓	✓	✓	–	✓	–	If Ca 8/20	N/A	28%	6%	66%	18/20 90%	0/20 (0%)	1 +ve SLN
3	–	✓ 0.5 mL *	✓ 37 MBq	✓	✓ 15/20	–	✓	–	If Ca 20/30	PN 2.2 PA 3.3	5%	0%	95%	PN: 14/15 (93.3%) PA: 20/20 (100%) Tc-99m: 20/20 (100%) ICG: 19/20 (95%)	2 (10%) vascular injury	No +ve LN
4	–	✓ 2 mL *	–	✓	✓ 15/31	–	✓ 18/31	✓ 13/31	✓ 30/31	2 (1–7)	62%	19%	19%	Overall: 67.7% Primary staging: 5/13 (38.5%) Secondary staging: 16/18 (88.9%)	1 (3.2%) superficial bladder injury	4 +ve SLN, 0 +ve non-SLN
5	–	✓ 0.5 mL *	✓ 37 MBq	✓	✓ 7/10	–	✓	–	If Ca 10/16	PN: 1.86 PA: 1.50	30%	30%	40%	100%	1 (10%) vascular injury	1/2 +ve SLN, 1/2 +ve non-SLN PA node (tracer did not migrate)
6	✓ 2 mL	–	✓ 1 mL	–	✓	–	✓	–	If Ca 3/11	N/A	60%	10%	30%	100%	N/A	1 patient had +ve SLN and non-SLN
7	–	✓0.5–1 mL ^†^	–	–	✓	✓ 3/5	✓	–	5/5	N/A	40%	20%	40%	100%	0 (0%)	All nodes negative
8	–	✓ 0.5–1 mL ^†^	–	✓	✓	–	✓	–	10/10	PN: 2.2 PA: 3.3	80%	20%	–	90%	1 (10%) vena cava injury	All nodes negative
9	✓	–	✓	✓ 25/35	✓ 25/35	✓ 10/35	✓	–	If Ca 13/35	2	79%	7%	14%	Overall: 71.4% Cortex: 40% IPL: 84%	0 (0%)	4 patients had ovarian torsion and SLN could not be identified; 3/3 non-SLN +ve also had SLN +ve
10	✓ 0.2–0.5 mL	–	✓ 20 MBq	✓	✓	–	✓	–	If Ca 6/21	PN: 1.86 PA: 1.50	67%	9%	24%	100%	N/A	1 patient had +ve SLN and non-SLN
11	–	–	–	–	–	✓	✓	–	–	N/A	64%	–	36%	100%	N/A	–

ICG = indocyanine green; Tc-99m = technetium-99m; IPL = infundibulopelvic ligament; UOL = utero-ovarian ligament; LND = lymphadenectomy; PA = para-aortic; PN = pelvic; FS = frozen section; Ca = cancer; CH40 = activated charcoal; * = 1.25mg/mL; ^†^ = 25 mg in 20cc water; +ve = positive.

**Table 4:** Ongoing trials in sentinel node mapping in early-stage ovarian cancer.

Study Name	No. of Participants	Type of Study	Condition	Tracer	Location of Injection	Primary Outcome	Expected Completion Date
MELISA (NCT05184140)	62	Prospective, single group assignment	Stage I and II ovarian cancer	Tc-99m and ICG	IPL and UOL	Detection rate of SLN technique	1 May 2023
SELLY (NCT03563781)	176	Prospective, single group assignment	Stage I ovarian cancer	Blue dye or ICG	IPL and UOL	1. Detection rate of SLN technique 2. No. of participants with procedure related adverse events	5 May 2023
MELISA II (NCT05937620)	62	Prospective, single group assignment	Stage I and II ovarian cancer	Tc-99m and ICG	N/A	Evaluation of the diagnostic efficiency of both tracers in SLN detection	October 2026
MagTrial (NCT05375526)	10	Prospective, single group assignment	Stage I and II ovarian cancer	MagTrace^®^ and Tc-99m	IPL and UOL	Assessment of the use of MagTrace^®^ as a tracer in SLN sampling	1 June 2024
NCT04051502	48	Prospective, observational	1. Ovarian cancer 2. Adnexal mass	ICG	1: Fallopian tube 2: 4 sites (dorsal and ventral side of the IPL and UOL) 3: IPL after removal of adnexal mass 4: IPL before removal of adnexal mass	Number and location of ovarian SLN visually identified after injection of ICG dye	August 2024
TRSGO-SLN-OO5 (NCT04714931)	200	Prospective, single group assignment	Stage I and II ovarian cancer	Blue dye or ICG	IPL and UOL	1. SLN localisation 2. SLN technique accuracy 3. PPV and NPV of SLN 4. Adverse events 5. Detection rate	1 April 2022
NCT05927818	100	Prospective, single group assignment	1. Adnexal mass 2. Stage I and II ovarian cancer	Sterile charcoal stain	IPL or mesovarium	Efficacy of SLN biopsy in highly suspicious adnexal masses	15 October 2025

Tc-99m = technetium-99m; ICG = indocyanine green; IPL = infundibulopelvic ligament; UOL = utero-ovarian ligament; SLN = sentinel lymph node; NIR = near-infrared camera; N/A = not available; PPV = positive predictive value; NPV = negative predictive value; −ve = negative.

## Data Availability

Data supporting the findings of this study are available from the corresponding author upon reasonable request.
